# The correlation study between the length and angle of ascending aortic and the incidence risk of acute type A aortic dissection

**DOI:** 10.3389/fcvm.2024.1375601

**Published:** 2024-03-25

**Authors:** Bowen Li, Xiangbin Meng, Chao Fu, Zhihao Yang, Xin Zhao

**Affiliations:** Department of Cardiovascular Surgery, Qilu Hospital of Shandong University, Jinan, China

**Keywords:** ATAAD, TAA, CTA, ascending aortic morphology, incidence risk of ATAAD

## Abstract

**Objective:**

This study utilized computed tomography angiography (CTA) to assess the risk of acute type A aortic dissection (ATAAD) by analyzing the imaging morphology indicators of the ascending aorta, along with the relevant risk factors associated with aortic dissection.

**Methods:**

The study utilized a retrospective observational research design. The population consisted of 172 patients who received treatment in the Department of Cardiothoracic Surgery at Qilu Hospital, Shandong University, from January 2018 to December 2022. The patients were divided into two groups: the ATAAD group (*n* = 97) and the thoracic aortic aneurysm group (TAA, *n* = 75). Demographic data and ascending aorta CTA measurements were collected from all patients. Single factor and multivariate logistic regression were employed to analyze the statistical differences in clinical data and ascending aorta CTA imaging morphology indicators between the two groups.

**Results:**

The variables were included in logistic multivariate analysis for further screening, indicating that the length of the ascending aorta (LAA) before ATAAD (OR = 3.365; 95% CI :1.742–6.500, *P<*0.001), ascending arch angle (asc-arch angle, OR = 0.902; 95% CI: 0.816–0.996, *P *= 0.042) and the maximum aortic diameter (MAD) before ATAAD, (OR = 0.614; 95% CI: 0.507–0.743, *P<*0.001) showed statistically significant differences.

**Conclusions:**

This study suggests that increased LAA and MAD, as well as a smaller asc-arch angle may be high-risk factors for the onset of ATAAD.

## Introduction

ATAAD is characterized by the disruption of the intima, leading to the separation of the aortic wall layers, followed by the formation of a true and false lumen (1). ATAAD is a rapidly progressing aortic disease with a high mortality rate (2, 3). Without timely intervention, mortality can increase by 1%–2% per hour within 48 h, reaching approximately 90% in 1 week (4). Population-based studies have shown that up to half of individuals affected with ATAAD die before reaching the hospital (5). However, a study revealed that out of 1,712 cases, only 140 patients arrived at the cardiovascular center within 8 h, indicating that many patients experience aortic dissection rupture (6).

At present, the most important approach to reducing the mortality rate of aortic diseases is through preventive surgical intervention (7). Therefore, a method is needed to screen patients at high incidence risk of ATAAD and enable timely surgical intervention. In the International Registry of ATAAD registry, over 60% of the patients had a MAD of less than 5.50 cm (8). The JACC 2022 guidelines (9) recommend preventive ascending aortic replacement surgery for aneurysms with a MAD greater than 5.00 cm in the general population. However, even with a lowered MAD threshold, 40% of patients with ATAAD still have a MAD of less than 4.50 cm (8).

Professor Poullis (10) studied the effect of aortic angle on ATAAD and found that as the curvature in the ascending aortic increases from straight to curved (90°), the force on the aortic wall increases by a factor of over 10. This is despite a normal aortic diameter, normal blood pressure and normal cardiac output. While reducing surgery thresholds may not necessarily resulting in a net benefit, it exposes a large low-risk population to significant surgical risk. Recent studies (11) have debated the clinical value of MAD measurements and focused on identifying new morphological predictors to enhance individualized incidence risk assessment for ATAAD (12–16). Our research aims to evaluate the effects of ascending aortic CTA morphology on the incidence risk of ATAAD.

## Participants and methods

### Patient selection

We utilized a retrospective observational research design. The study included a population of 172 patients who received treatment in the Department of Cardiothoracic Surgery at Qilu Hospital, Shandong University from January 2018 to December 2022. This study was approved by the Institutional Review Board of Qilu Hospital at Shandong University (KYLL-202307-032). Due to the retrospective nature of the study, the requirement for obtaining informed consent was waived. The study population was then divided into two groups: the ATAAD group (*n* = 97); and the TAA group, which included patients who underwent elective surgeries for TAA (*n* = 75). For ATAAD patients, CT scans were obtained at the time of acute presentation, while for TAA patients, the scans were taken during the preoperative workup. The exclusion criteria included missing preoperative CT data, ascending aortic wall hematoma, previous history of cardiovascular surgery, abnormal development of the aorta, and iatrogenic and connective tissue aortic lesions. Primary aortic stenosis, aortic regurgitation, and congenital aortic valve disease (including bicuspid aortic valve) may lead to abnormal aortic hemodynamics, which can also result in morphological changes in the ascending aorta. This study aims to evaluate the occurrence of ATAAD using aortic morphology, a directly observable indicator. The reasons for the changes in aortic morphology were not considered or distinguished. Therefore, patients with concomitant aortic valve disease were not excluded.

### Definition and measuring methods of aortic morphology

Aortic measurements were obtained from preoperative CTA (Philips intellispace PACS Enterprise v4.4) for patients with ATAAD and TAA. All measurements of the ascending aorta are manually completed using graphical measurement tools in the post-processing SYNGO.VIA application software. We used a more standardized method of measurement, such as projection in the coronal and axial plane for all patients, instead of rotating the 3-dimensional image to find a MAD (16). The ascending aorta was defined as the segment between the sinotubular junction and the origin of the brachiocephalic trunk. For ATAAD, measurements were intra-luminal, including both true and false lumens. To minimize inter-rater variability, the same trained clinician performed all measurements.

LAA was measured from the midpoint of the sinotubular junction plane to the midpoint of the plane of origin of the brachiocephalic trunk artery. The MAD of the ascending aorta referred to the maximum diameter of the aorta between the sinotubular junction and the starting plane of the innominate artery. Aortic diameter and length refer to measurement methods in previous studies (7).

**Figure 1:**
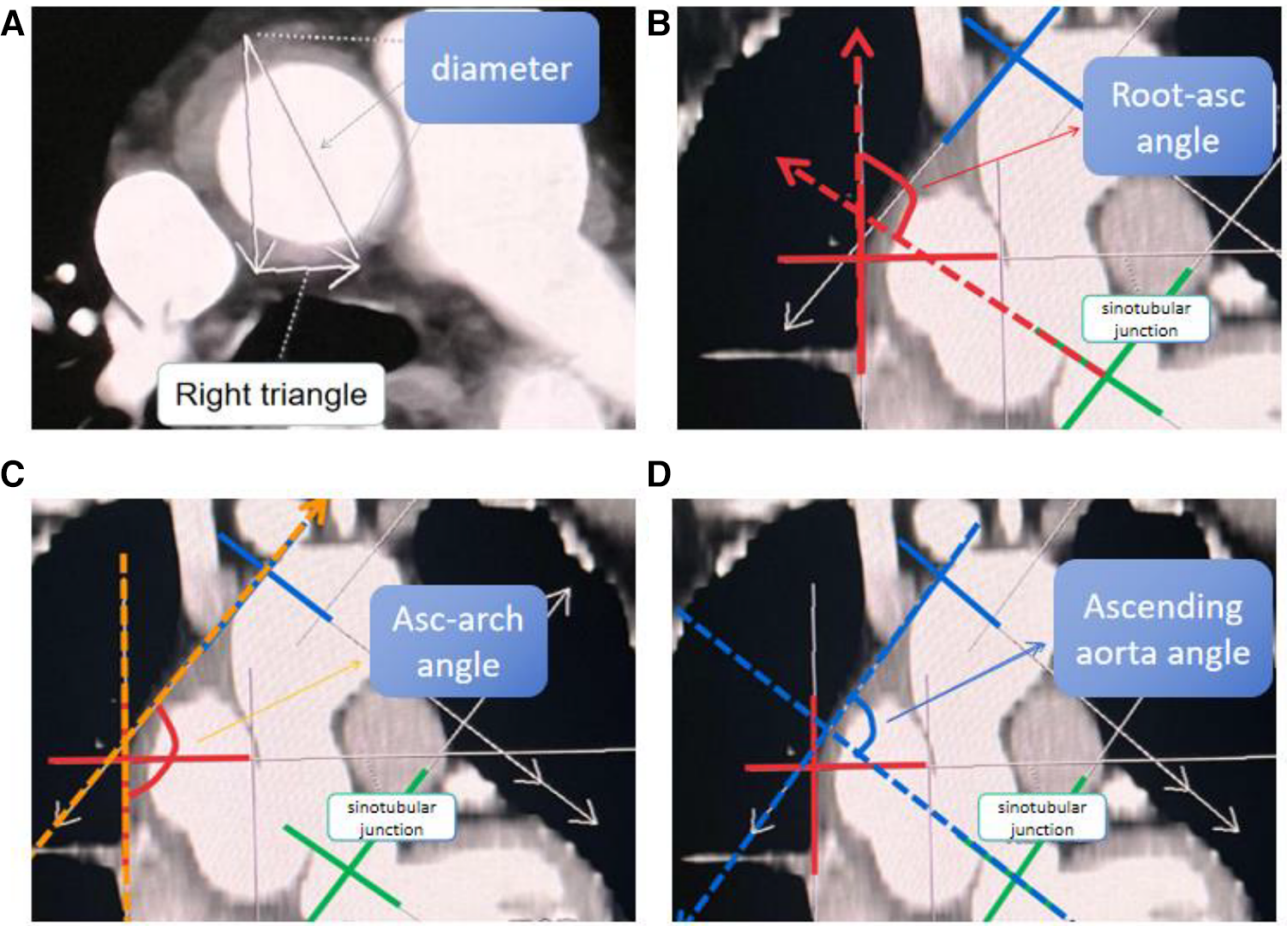
Measurement of the MAD of the ascending aorta and measurement of the “three angles” of the aorta. (**A**) Diameter of Aortic: the MAD of the aorta between the sinotubular junction and the starting plane of the innominate artery. (**B**) The root-asc angle: the angle between the vertical line of the sinotubular junction plane and the vertical line of the aortic cross-section at the midpoint of the main pulmonary artery. (**C**) The asc-arch angle: the angle between the vertical line of the aortic cross-section at the level of the brachiocephalic artery and the vertical line of the aortic cross-section at the midpoint of the main pulmonary artery. (**D**) Ascending aorta angle: the angle between the vertical line of the ascending aorta's junction plane and the vertical line of the aortic transverse plane at the level of the brachiocephalic artery.

**Figure 2:**
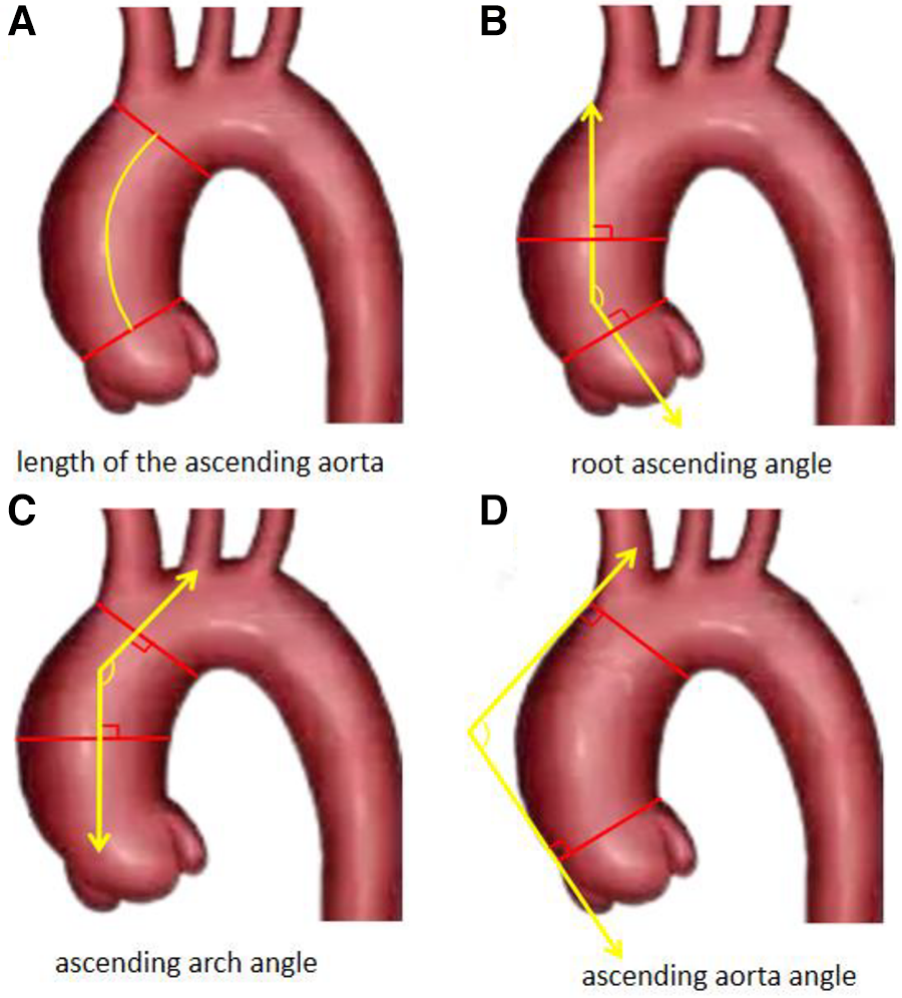
The diagrammatic sketch of the LAA and the “three angles” of the aorta.

The ascending aortic angle is defined as the angle between the vertical line of the ascending aorta's junction plane and the vertical line of the aortic transverse plane at the level of the brachiocephalic artery. The root ascending angle (root-asc angle) is determined by the angle between the vertical line of the sinotubular junction plane and the vertical line of the aortic cross-section at the midpoint of the main pulmonary artery. Similarly, the asc-arch angle is defined as the angle between the vertical line of the aortic cross-section at the level of the brachiocephalic artery and the vertical line of the aortic cross-section at the midpoint of the main pulmonary artery. Aortic related angles refer to measurement methods in previous studies (14), as shown in Figures 1, 2. We have decided to use rylski's criteria (17) to infer the morphological indicators of the aorta before the occurrence of ATAAD. After the occurrence of ATAAD, the MAD of the ascending aorta increased by 30%, and the LAA increased by 5%.

### Statistical analyses

Statistical analysis of the data was performed using commercially or freely available software (SPSS V.25, IBM, Armonk, New York, USA; or R Statistics, the R Foundation, Vienna, Austria). Continuous data were presented as mean ± SD (or median and 25%, 75% in the presence of skewedness) and were compared using the student t-test or Mann-Whitney U-test. Categorical data were expressed as frequencies and percentages and were compared using the *χ*^2^ test. Single factor and multivariate logistic regression were used to analyze the statistical differences in clinical data and ascending aorta CTA imaging morphology indicators between the two groups of patients. *P *< 0.05 was considered statistically significant. Use the “pwr” package in the R language to analyze the necessary sample size for the study. The preset effect size for the independent sample t-test difference analysis is d = 0.5, and the preset statistical test size is 1- β = 0.8, with a significance level of a = 0.05. The results show that each group requires a minimum of 64 study subjects, and the existing database already meets this sample size. Receiver operating characteristic (ROC) analysis was used to assess the accuracy of the three measurements, with the calculated area under the curve (AUC) indicating the discrimination quality.

## Results

The ATAAD group consisted of 69 males and 28 females, with a median age of 53.37 years. In the TAA group, there were 55 males and 20 females, with a median age of 53.99 years. In the ATTAD group, 65 people (67%) had a history of hypertension, while in the TAA group, 26 people (34.7%) had a history of hypertension. The history of hypertension was found to be statistically significant in the single-factor analysis. There were no significant statistical differences in age, sex, height, weight, BMI, smoking, and drinking history between the ATAAD group and the TAA group, as shown in Table 1.

**Table 1:** The univariate analysis of baseline data of ATAAD and TAA groups.

	ATAAD group (*n* = 97)	TAA group (*n* = 75)	*P*-value
Age (years)	53.37 ± 12.17	53.99 ± 11.45	0.736
Male gender	69 (71.1%)	55 (73.3%)	0.750
Height (m)	1.72 (1.65, 1.75)	1.70 (1.62, 1.75)	0.694
Weight (kg)	75.14 ± 15.02	71.56 ± 12.35	0.096
MBI (kg/m^2^)	25.39 (22.77, 29.03)	24.80 (22.65, 26.95)	0.098
Drinking (%)	48 (49.5%)	36 (48.0%)	0.847
Smoking (%)	36 (37.1%)	32 (42.7%)	0.460
Hypertension	65 (67.0%)	26 (34.7%)	<0.001

The average LAA before dissection in the ATAAD group was 10.48 ± 1.48 cm, while in the TAA group, the average LAA before dissection was 9.54 ± 1.67 cm. The average root-asc angle in the ATAAD group was 129.34° ± 8.07°, while the average root-asc angle in the TAA group was 134.08° ± 9.98°. The average ascending aortic angle in the ATAAD group was 82.77° ± 10.53°, while in the TAA group it was 96.36° ± 13.93°. The median MAD of the ascending aorta before dissection in the ATAAD group was 3.78 cm (Q1–Q3, 3.40–4.15 cm), while in the TAA group it was 5.61 cm (Q1–Q3, 5.17–6.02 cm). The median asc-arch angle of the ATAAD group was 133° (Q1–Q3, 127°–139°), while in the TAA group it was 143° (Q1–Q3, 136°–150°). In the single-factor analysis of aortic CT morphology, both the MAD and LAA before dissection, the root-asc angle, the asc-arch angle, and the ascending aorta angle are statistically significant, as shown in Table 2.

**Table 2:** The data of univariate analysis of imaging measurement data of ascending aorta of ATAAD and TAA groups.

	ATAAD group (*n* = 97)	TAA group (*n* = 75)	P
MAD before ATAAD (cm)	3.78 (3.40, 4.15)	5.61 (5.17, 6.02)	<0.001
LAA before ATAAD (cm)	10.48 ± 1.48	9.54 ± 1.67	<0.001
Root-asc Angle (°)	129.34 ± 8.07	134.08 ± 9.98	0.001
Asc-arch angle (°)	133 (127, 139)	143 (136, 150)	<0.001
Ascending aorta angle (°)	82.77 ± 10.53	96.36 ± 13.93	<0.001

**Figure 3:**
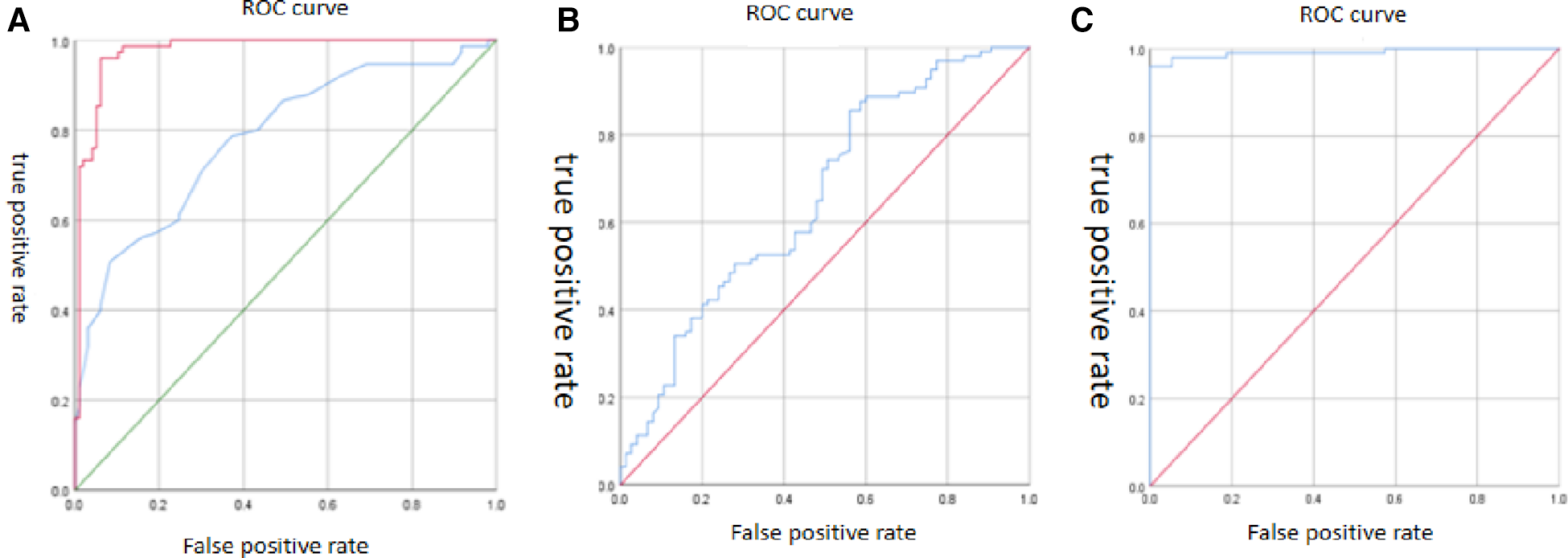
Receiver operating characteristic curve. (**A**) The area under the curve of MAD of the ascending aorta (red curve) and asc-arch angle (blue curve) are 0.976 and 0.782. (**B**) The area under the curve of the LAA before ATAAD is 0.658. (**C**) The area under the curve of the logistic multiple factor analysis model is 0.991.

The univariate analysis revealed statistically significant differences between the ATAAD group and TAA group in variables such as root-asc angle, asc-arch angle, ascending aorta angle, hypertension history, the MAD before dissection, and the LAA before dissection. Further screening through logistic multivariate analysis included the above variables and showed statistically significant differences in variables such as the LAA before dissection (OR = 3.365; 95% CI: 1.742–6.500, *P* < 0.001), asc-arch angle (OR = 0.902; 95% CI: 0.816–0.996, *P* = 0.042), and MAD of the ascending aorta before dissection (OR = 0.614; 95% CI: 0.507–0.743, *P* < 0.001), as shown in Table 3. The area under the curve for the MAD of the ascending aorta, asc-arch angle, and ascending aorta length before ATAAD are 0.976, 0.782, and 0.658, respectively, as shown in Figure 3.

**Table 3:** The logistic multi-factor analysis of incidence risk of ATAAD vs. TAA group.

	B	Wald	P	OR	95% CI	
					LL	UL
Asc-arch angle (°)	−0.103	4.128	0.042	0.902	0.816	0.996
MAD before ATAAD (cm)	−0.488	25.011	<0.001	0.614	0.507	0.743
LAA before ATAAD (cm)	1.213	13.048	<0.001	3.365	1.742	6.500

## Discussions

ATAAD is a highly dangerous condition with a high mortality rate, even with surgical intervention. Prophylactic ascending aortic replacement for ATAAD is often considered to reduce the risk of aortic dissection. However, elective ascending aortic replacement carries its own risks of major morbidity and mortality. Therefore, accurately assessing the incidence risk of ATAAD is crucial for optimizing clinical decision-making, minimizing morbidity, and maximizing patient survival (18).

It is crucial to accurately infer the morphological indicators before the onset of ATAAD, as previous studies have revealed changes in the geometric shape of the aorta at the moment of ATAAD. Rylski (17) established a database of aortic diameter before dissection based on a cohort of 343 ATAAD patients, measured after dissection, minus an average diameter increase rate of 30% (based on the results of a study on changes in human aortic dissection). Mansour (19) found in their study that the diameter of the aorta before dissection was, on average, 7 mm smaller than that measured after dissection (with an average diameter increase rate of 20% after dissection). Other studies have reported an increase of 20%–30% in the MAD, a 5% increase in the LAA, and a 2.7% increase in the LAA reported by Wu (20).

International guidelines for the registration of dissection pointed out that 60% of cases have a MAD of less than 5.50 cm before dissection, and 40% have a MAD less than 4.50 cm (8). Therefore, it is likely inappropriate to use diameter as a predictive indicator for ATAAD.

Other indicators need to be explored. Previous studies have demonstrated that the LAA increases with age (21). In this study, the LAA was found to be a highly sensitive and specific factor for evaluating the incidence of ATAAD. Previous studies have also indicated that the longitudinal displacement of the aorta increases, resulting in a 50% increase in longitudinal stress at approximately 2.00 cm on the junction of the great curved side of the aorta with the sinus tube (22). The diameter of ATAAD increased by 18% before and after dissection, while the length of the aorta increased by 2.7%. It has been confirmed that aging of the aorta is associated the fracture and rupture of elastin fibers, leading to a decrease of vascular compliance (23). The prolongation itself may indicate thinning of the aortic wall and rupture of elastin fibers. Taking into account previous aortic size, the time interval, age, and gender, the sudden increase in aortic length caused by dissection itself approaches zero. The relative stability of the length of the ascending aorta in relation to aortic dissection helps determine an appropriate intervention threshold (12, 14, 17, 20). At the level of the ascending aorta, longitudinal compliance is asymmetric (24), and with an increase in ascending aortic length, the side with the great curve bears more longitudinal force. Therefore, in the presence aging of the patient's aorta and the impact of high-speed blood flow, the side with the great curve is prone to rupture. Among patients without Marfan syndrome, the majority of ATAAD occur at a relatively older age. This study revealed a median age of 54 years, suggesting advanced age as a potential risk factor for ATAAD.

This study clarifies that in patients with a larger angle of the ascending aorta but a larger aortic diameter of TAA, the shear force on the aorta is relatively smaller. The larger angle of the aorta makes it less prone to aortic dissection. If the angle of the ascending aorta is narrow, turbulent flow occurs, resulting in greater wall shear stress (16). It can be hypothesized that, similar to the LAA, the asc-arch angle is not affected by significant acute changes during ATAAD development. Therefore, narrowing of the asc-arch angle is likely to be a pre-existing feature of the aortic geometry that predisposes to ATAAD (14). There was significant statistical difference in the asc-arch angle between the ATAAD group and the TAA group. This implies that even if the diameter of patients with TAA is much larger than that of patients with ATAAD, aortic dissection did not occur due to the sufficiently large angle of the related aorta, resulting in reduced blood flow shear force; The reason why aortic dissection occurs in patients whose diameter is similar to that of normal individuals might be that the angle of ascending aortic is small, leading to increased shear force.

## Conclusions

This study suggests that increased the LAA and the MAD, as well as a smaller asc-arch angle may be high-risk factors for the onset of ATAAD. This study suggests that an increased LAA and MAD, along with a smaller asc-arch angle, may be high-risk factors for the onset of ATAAD.

### Limitation

This is a single-center research study that lacks multicenter validation, and there may be minor subjective errors in the measurement of morphological data. Another limitation is the lack of longitudinal follow-up, which does not accurately identify a “hinge” point.

## Data Availability

The raw data supporting the conclusions of this article will be made available by the authors, without undue reservation.
